# A Court Appointment

**Published:** 1897-08-07

**Authors:** 


### A COURT APPOINTMENT.

There are certain people who carp at everything they do not understand. We need, then, feel no great surprise at the criticisms which have been evoked by the appointment of Mr. Fripp as Surgeon in Ordinary to H.R.H. the Prince of Wales along with Sir William MacCormac. Mr. Fripp is a young man; nevertheless, it is hardly fair to speak of him as one who has "barely passed out of his 'teens' in surgery." He qualified in 1888; he is now a Fellow of the Royal College of Surgeons; he has gone through the various posts taken by the more distinguished students, such as Clinical Assistant, Resident Obstetric, and House Physician; he was a Prosector to the Royal College of Surgeons; he has become the Senior Demonstrator of Anatomy at the Guy's Hospital Medical School, and he is Assistant Surgeon to the hospital. We mention these points, not in praise of Mr. Fripp, but to show that the Prince of Wales, in making this appointment, did not exercise his undoubted right in an arbitrary manner, but made his choice from among those who had already given proof of their capacity. We think, however, that as people have chosen to whet their critical faculties on this appointment, the full truth should be known, and that it should be generally understood that this is but another instance of what people will, perhaps, some time recognise, that when the Prince of Wales takes independent action, he is moved thereto both by knowledge and by kindness of heart. At a time of great trial, when the Royal house was plunged in grief, and when a young Prince, who a week before had seemed to have every prospect of ending his days as King of England, was struck down by a fatal disease, then it was that Mr. Fripp came under the notice of the Prince of Wales. Are we to imagine that when, at such a juncture, it became necessary to increase the band of watchers the physicians in charge sent down the first man they came across? On the contrary, the very fact of being chosen for such work was in itself proof of the high estimation in which he was already held. The point, however, is this, that it was in those sad days that the Prince of Wales got to know his man. Those who know how Prince Eddy was watched and tended will recognise that this appointment is largely the outcome of personal knowledge. The Prince saw and was grateful, and we feel sure the public will fully endorse an act which, as we have already said, shows both knowledge and kindness of heart.

